# Hydrodeoxygenation of Lignin-Based Compounds over Ruthenium Catalysts Based on Sulfonated Porous Aromatic Frameworks

**DOI:** 10.3390/polym15234618

**Published:** 2023-12-04

**Authors:** Maria A. Bazhenova, Leonid A. Kulikov, Daria A. Makeeva, Anton L. Maximov, Eduard A. Karakhanov

**Affiliations:** 1 Department of Petroleum Chemistry and Organic Catalysis, Lomonosov Moscow State University, Moscow 119991, Russia; bazhenova.maria.a@yandex.ru (M.A.B.); d-makeeva95@yandex.ru (D.A.M.); max@ips.ac.ru (A.L.M.); kar@petrol.chem.msu.ru (E.A.K.); 2 Institute of Petrochemical Synthesis RAS, Moscow 119991, Russia

**Keywords:** hydrodeoxygenation, porous aromatic frameworks, ruthenium catalysts, guaiacol, lignin

## Abstract

Bifunctional catalysts are a major type of heterogeneous catalytic systems that have been widely investigated for biomass upgrading. In this work, Ru-catalysts based on sulfonated porous aromatic frameworks (PAFs) were used in the hydrodeoxygenation (HDO) of lignin-derived compounds: guaiacol, veratrole, and catechol. The relationship between the activity of metal nanoparticles and the content of acid sites in synthesized catalysts was studied. Herein, their synergy was demonstrated in the Ru-PAF-30-SO_3_H/5-COD catalyst. The results revealed that this catalytic system promoted partial hydrogenation of lignin-based compounds to ketones without any further transformations. The design of the Ru-PAF-30-SO_3_H/5-COD catalytic system opens a promising route to the selective conversion of lignin model compounds to cyclohexanone.

## 1. Introduction

Nowadays, increasing attention is being paid to the development of technologies that ensure carbon neutral production of fuels and chemical compounds from renewable sources [1,2,3,4,5]. An example of such raw material is lignin, a complex and disordered polymer of aromatic nature, present in plants in significant quantities (about 25%). Lignin consists of phenylpropane derivatives containing hydroxyl and methoxy functional groups linked together by ether or carbon-carbon bonds. This structure allows us to consider lignin as a source of various value-added products: phenolic aldehydes [6], ketones [7], acids [8], cresol, and aromatic and saturated hydrocarbons [9,10,11]. The product of lignin pyrolysis, bio-oil, is a dark brown liquid consisting of hundreds of highly oxygenated organic compounds. However, the direct use of the lignin-derived bio-oil in petrochemical processing is complicated due to some issues: increased acidity and corrosiveness, high viscosity, low calorific value, and a tendency to reverse polymerization. One of the solutions is hydrodeoxygenation (HDO) of bio-oil, during which hydrogenation and removal of unsaturated and oxygen-containing compounds occurs. Thus, the development of technologies and catalytic systems for the processing of lignin-derived bio-oil is an urgent task.

Traditional catalysts of hydrodesulfurization (HDS) processes—Mo, Ni, and Co sulfides deposited on γ-alumina—can be applied for bio-oil HDO [12,13]. However, their use is accompanied by several problems, such as the formation of oxides and further deactivation of catalysts by water contained in bio-oil (up to 30%) and generated in the HDO process [14,15]. To prevent deactivation of sulfide catalysts, their sulfide form must be maintained during HDO, which is often achieved by adding an appropriate source of sulfur to the feed. This may lead, in its turn, to the emergence of technological difficulties and contamination of products. Catalysts based on noble metals such as Ru, Pt, Pd, and Rh can be used to overcome disadvantages of the traditional catalysts. First, metal loadings in such systems are usually much lower compared to those used in traditional metal sulfide catalysts, and they are also expected to be more stable in the presence of oxygen-containing compounds. Noble metal catalysts have a remarkable ability to activate and split hydrogen under mild conditions [16,17,18], which allows us to avoid high reaction temperatures and reduce the rate of coke deposition.

In general, catalysts based on supports with acidic properties are more active in HDO reactions. For example, it was shown that the presence of Brønsted acids leads to the significant increase in the yield of cyclohexane, the product of complete guaiacol HDO, from 20.1% for Ru/C to 99.9% for Ru/C+H_3_PO_4_ [19]. Similar effects were also noted for heterogeneous catalytic systems with grafted acid groups [20]. Thus, both Pd-PAF-30 and Pd-PAF-30-SO_3_H catalysts were tested in the vanillin hydrodeoxygenation [21]. It was demonstrated that modification of PAF-30 with sulfo groups greatly enhanced the activity of the catalyst in deoxygenation. The increase in deoxygenation activity of the catalysts investigated in HDO of guaiacol with the increase in concentration of acid sites was also observed [22]. The proximity of metal active sites and acid groups of support also may have additional synergistic effects on the catalytic activity [20,23,24,25], such as, for example, enhancement of the overall reaction rate [26,27].

A plethora of research on hydrodeoxygenation in the presence of noble metals supported on inorganic materials (silica, alumina, titanium oxide [28,29], and zeolites [30]) is presented in the literature. Catalysts based on inorganic supports demonstrate high catalytic activity, but even so, many of them are unstable under acidic and basic conditions and prone to deactivation resulted from coking. Only a small number of works are devoted to the study of HDO catalysts based on porous organic polymers (POPs). Despite this, their high specific surface areas and developed porosity alongside high thermal and mechanical stability makes them attractive in heterogeneous catalysis. In addition, the possibility to incorporate different functional groups in the structures of POPs allows us to tune the activity and selectivity of obtained catalytic system.

Recently, we reported HDO of bio-oil components using Pt and Ru catalysts based on Porous Aromatic Frameworks (PAFs), polymers with rigid structure consisting of interconnected aromatic rings [21,31,32,33,34]. We have shown that the presence of -SO_3_H groups increases the yield of cycloalkanes in HDO of guaiacol and its derivatives [35]. The purpose of the present study was to investigate the effect of the content and the mutual arrangement of metal and acid sites on the process of hydrodeoxygenation of guaiacol, veratrole, and catechol. To achieve this goal, two approaches to the synthesis of nanoparticles in the porous structure of PAFs with different content of acid sites were used: by wetness impregnation with (A) only RuCl_3_ solution and (B) with RuCl_3_ solution in the presence of 1,5-cyclooctadiene, which is able to complex with the metal and facilitate its incorporation into the pores of the support.

## 2. Materials and Methods

### 2.1. The Materials

The following reagents were used in this work: ethanol (high-purity grade Component-Reactiv); isopropyl alcohol (high-purity grade, Component-Reactiv); 1,5-cyclooctadiene (≥99%, Sigma-Aldrich, St. Louis, MO, USA), tetrahydrofuran (high-purity grade, Component-Reactiv); diethyl ether (high-purity grade, Component-Reactiv); dichloromethane (high-purity grade, Component-Reactiv); acetic acid (high-purity grade Ruskhim, Staraya Kupavna, Moscow region, Russia); chlorosulfonic acid (99%, Sigma-Aldrich, Darmstadt, Germany); sodium hydroxide (Reakhim, 99%); sodium borohydride (98%, Sigma-Aldrich, Germany); 2-methoxyphenol (99%, Sigma-Aldrich, Wuxi, China); 1,2-dimethoxybenzene (99%, Sigma-Aldrich, China); and 1,2-dihydroxybenzene (99%, Sigma-Aldrich, China). Component-Reactiv reagents were purchased from Moscow, Russia.

### 2.2. Synthesis

Porous aromatic framework PAF-30 was prepared by Suzuki cross-coupling reaction between tetrakis-(*p*-bromophenyl)methane and 4,4′-biphenyldiboronic acid according to the published procedure [36].

PAF-30-SO_3_H/X (X = 2.5, 5, 7.5). Sulfonation of PAF-30 was carried out in a 50 mL one-neck flask equipped with a magnetic stir bar. The suspension of PAF-30 (500 mg) in dichloromethane (25 mL) was cooled down to 0 °C, and chlorosulfonic acid (100, 167, or 250 μL for PAF-30-SO_3_H/2.5, PAF-30-SO_3_H/5, and PAF-30-SO_3_H/7.5, respectively) was added dropwise afterwards. The resulting mixture was stirred at room temperature for 24 h. After completion of the reaction, the suspension was poured into ice, then the solid product was filtered, washed with water, THF, and diethyl ether, and dried in a vacuum. Samples were named according to the theoretical sulfur content in synthesized porous polymers: 2.5 wt.% in PAF-30-SO_3_H/2.5, 5 wt.% in PAF-30-SO_3_H/5, and 7.5 wt.% in PAF-30-SO_3_H/7.5.

Ru-PAF-30-SO_3_H/X (X = 2.5, 5, 7.5) catalysts. RuCl_3_ (10.8 mg, 0.052 mmol) and 10 mL of ethanol were placed in a 25 mL one-neck flask equipped with a magnetic stir bar. After dissolution of all RuCl_3_, 100 mg of PAF-30-SO_3_H/X was added to the mixture and the resulting suspension was left to stir for 24 h. It was then cooled to 0 °C, and 15 mL of a cooled NaBH_4_ solution (400 mg, 21 mmol) in a water/methanol system (1:1) was added to the suspension dropwise under vigorous stirring; the reaction was then conducted for another 24 h. The resulting gray precipitate was filtered, washed with ethanol (50 mL), acetic acid (50 mL), water (2 × 50 mL), and ethanol (50 mL), and dried in a vacuum for 4 h. Acetic acid was used to remove the residual Na^+^ cations.

Ru-PAF-30-SO_3_H/X-COD (X = 2.5, 5, 7.5) catalysts. The procedure was the same as the one described above, except for the addition of 1,5-cyclooctadiene (0.125 mL) at the stage of the preparation of RuCl_3_ solution.

### 2.3. Characterization

Nitrogen adsorption isotherms were recorded on a Micromeritics Gemini VII 2390 instrument (Micromeritics, Norcross, GA, USA). All samples were degassed at 120 °C for 8 h before analysis. The surface area (S_BET_) was calculated using the Brunauer–Emmett–Teller (BET) method based on adsorption data in the relative pressure range P/P_0_ = 0.05-0.25. The total pore volume (V_tot_) was determined by the amount of nitrogen adsorbed at a relative pressure of P/P_0_ = 0.965.

IR spectra were recorded with a Nicolet IR200 (Thermo Scientific, Waltham, MA, USA) instrument using multiple distortion of the total internal reflection method with multi-reflection HATR accessories, containing a 45° ZnSe crystal for different wavelengths with a resolution of 4 cm^−1^ in the range of 4000–500 cm^−1^. Spectra of the catalysts after the reaction were obtained by pressing the material into a tablet with KBr. All spectra were taken by an average of 100 scans.

Chemical composition (compositional weight percentage of carbon, hydrogen, and sulfur) was determined with CHNS elemental analyzer Thermo Flash 2000 in Center for Collective Usage ‘Analytical Center for the Problems of Deep Refining of Oil and Petrochemistry’ at the A.V. Topchiev Institute of Petrochemical Synthesis, RAS.

The ruthenium concentrations in the catalysts were measured by inductively coupled plasma atomic emission spectrometry (ICP-AES) on a SHIMADZU ICPE-9000 spectrometer.

Transmission electron microscopy (TEM) images were obtained using a JEOL JEM-2100/Cs/GIF microscope (JEOL, Tokyo, Japan) with a 0.19 nm lattice fringe resolution and an accelerating voltage of 200 kV. The processing of the micrographs and the calculation of the average particle size were conducted using the ImageJ 1.54g software program. The analysis was performed in the center “Materials Science and Metallurgy” of NUST MISiS.

XPS studies were performed on a PHI VersaProbe II 5000 instrument using excitation with Al Kα X-ray radiation at 1486.6 eV. The calibration of photoelectron peaks was based on the C1s line with a binding energy of 284.5 eV. The transmission energy of the energy analyzer was 160 eV (survey scans) and 23.5 eV (individual lines). Deconvolution of palladium high-resolution spectra was performed using CasaXPS v. 2.3.19PR1.0 software. The analysis was performed in the center “Materials Science and Metallurgy” of NUST MISiS.

The acidity measurement was carried out by acid-base titration. The acid catalyst was dispersed in a standard solution of NaCl (0.01 mol/L), and then in a standard solution of NaOH (0.01 mol/L) as titrant. Acid-base potentiometric titration was carried out using a PH-009(II) pH meter with the following characteristics: pH measurement ranges from 0.00 to 14.00; resolution 0.01 pH; accuracy ±0.01 pH.

Acidity was calculated by the following Equation (1):

(1) $$Acidity\;\left(\frac{mmol}{g}\right)=\frac{C_{NaOH}\times V_{NaOH}}{m_{PAF}}$$

### 2.4. Reaction Procedure and Product Analysis

Hydrodeoxygenation of lignin-based compounds was carried out in a stainless-steel batch reactor equipped with magnetic stirrer. To begin, 5 mg of the catalyst and 0.38 mol of a substrate in 500 µL of water were loaded in the reactor. Reactions were carried out for 2 h at a hydrogen pressure of 30 bar and a temperature of 250 °C. After completion of the reaction, the autoclave was cooled to room temperature and depressurized. Reaction products were analysed by gas chromatography. All experiments were performed at least twice; the experimental error did not exceed 5%.

The analysis of reaction products was carried out by gas chromatography-mass spectroscopy on a Leco Pegasus^®^ GC-HRT 4D instrument with parallel detection of components on a time-of-flight mass spectrometer and a flame ionization detector. The analysis was carried out using equipment purchased at the support of the Moscow University Development Program. The structure of the components was determined by analyzing the mass spectra using the NIST v.2.3 library dated 4 May 2017.

The conversion was calculated using the following Formula (2):

(2) $$\eta =\frac{C_{substrateinintial}-C_{substrateresidual}}{C_{substrateinintial}}\times 100\%$$

The yield of reaction product was calculated by the next Formula (3):

(3) $$w_{\;i}=\frac{c_{\;i}}{\sum c}\times 100\%$$

## 3. Results and Discussion

### 3.1. Characterization of the Materials

**Figure 1:**
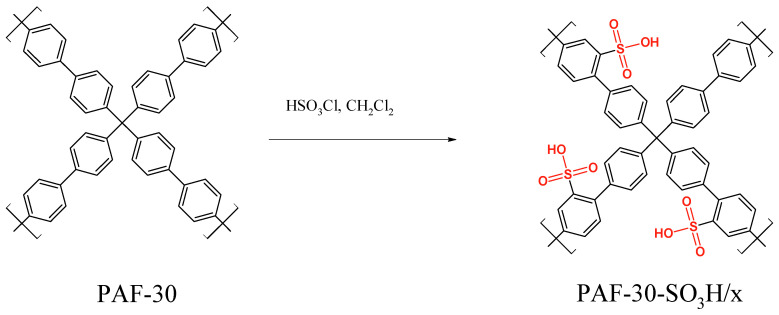
Synthesis of PAF-30-SO_3_H/x, where x = 2.5, 5 or 7.5.

The initial polymer PAF-30 was synthesized from tetrakis-(*p*-bromophenyl)methane and 4,4-diphenyldiboric acid via Suzuki cross-coupling. PAF-30 was then modified with -SO_3_H groups by treating it with a solution of chlorosulfonic acid (100, 167 or 250 µL) in dichloromethane, resulting in PAF-30-SO_3_H/2.5, PAF-30-SO_3_H/5, and PAF-30-SO_3_H/7.5 materials containing 2.5, 5, and 7.5 wt. % of sulfur, respectively, according to theoretical calculations (Figure 1).

**Figure 2:**
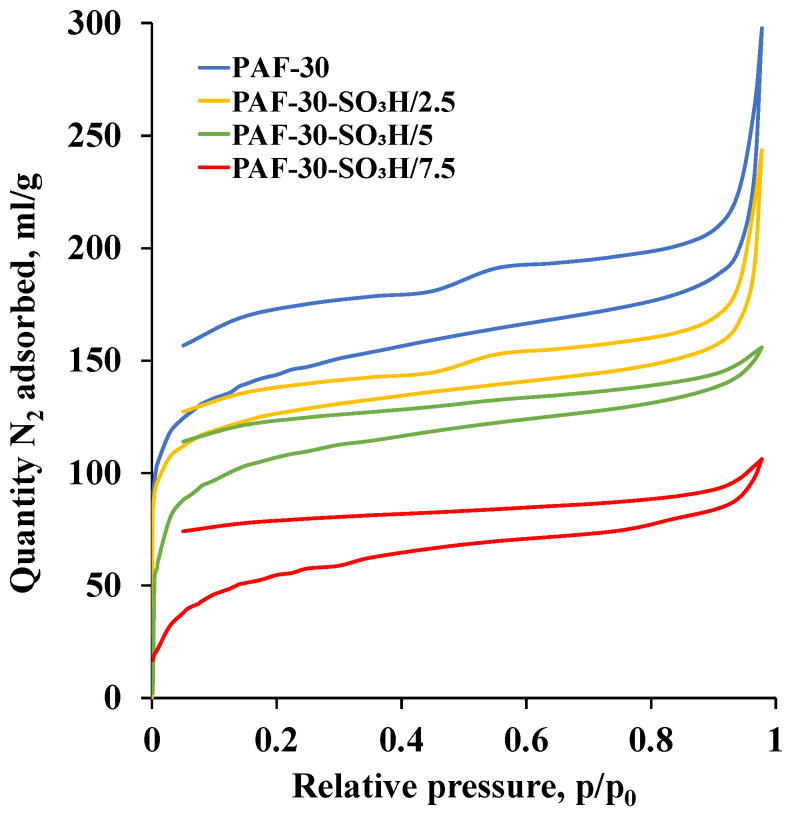
N_2_ adsorption isotherms for PAF-30, PAF-30-SO_3_H/2.5, PAF-30-SO_3_H/5, and PAF-30-SO_3_H/7.5.

According to the data of low-temperature N_2_ adsorption-desorption (Figure 2), the adsorption isotherm of PAF-30 exhibits a steep increase at low p/p_0_ values. The hysteresis between adsorption and desorption branches indicates that PAFs consist not only of micro- but also of mesopores. For isotherms of sulfonated materials, the decrease in quantity of adsorbed nitrogen with an increase in sulfur content should be noted, which may indicate the blockage of porous structure with sulfo groups resulting in limited nitrogen diffusion. Introduction of functional groups also led to the reduction of total pore volume (Table 1). The acidity of sulfonated polymers, determined by acid-base titration, increased from 0 for PAF-30 to 2.34 mmol/g for PAF-30-SO_3_H/7.5, which also confirms the introduction of various amounts acidic -SO_3_H groups into the structure of frameworks.

**Figure 3:**
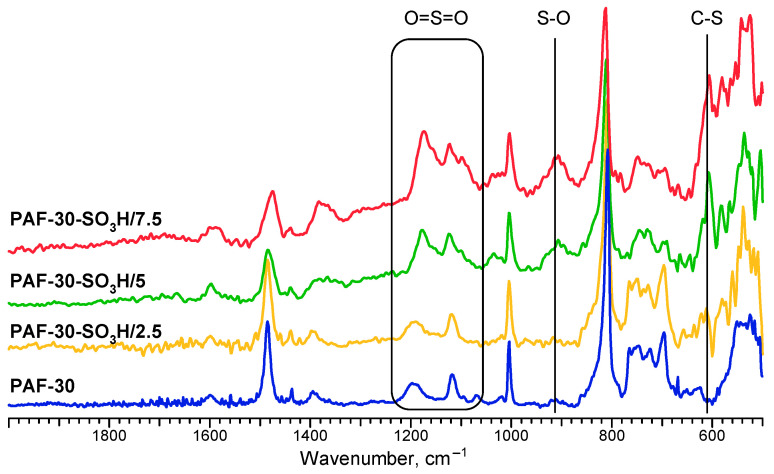
FTIR spectra of synthesized polymers.

Incorporation of the sulfo groups in the material was additionally confirmed by the appearance of novel absorption bands in the FTIR spectrum (Figure 3). New signals that appeared at 1193 and 1095 cm^−1^ can be attributed to the O=S=O symmetric and asymmetric stretching modes, respectively. Meanwhile, the bands at 631 and 1030 cm^−1^ correspond to the C-S and S-O stretching vibrations, respectively [37].

The presence of sulfo groups in the materials was confirmed using XPS. According to the analysis of survey spectra (Figure S1), the materials contain 1.0–3.9 at. % of sulfur (Table S1), and high-resolution XPS spectra of S2p region (Figure S2) contain peaks with binding energies of 169.6 eV and 168.4 eV, which correspond to 2p_1/2_ and 2p_3/2_ states of -SO_3_H groups, respectively [38,39].

Ruthenium catalysts were synthesized by incipient wetness impregnation method using RuCl_3_ solution—Ru-PAF-30-SO_3_H/2.5, Ru-PAF-30-SO_3_H/5, and Ru-PAF-30-SO_3_H/7.5 (Series A)—and with RuCl_3_ in the presence of 1,5-cyclooctadiene—Ru-PAF-30-SO_3_H/2.5-COD, Ru-PAF-30-SO_3_H/5-COD, and Ru-PAF-30-SO_3_H/7.5-COD (Series B). It is known that RuCl_3_ in an aqueous solution is presented in the form of sufficiently bulky oxygen-bridged complexes [40]. Due to their size, their diffusion into the pore space of the support may be impeded and, therefore, it may be difficult for them to reach acidic sulfo groups. The presence of relatively labile ligands like COD (1,5-cyclooctadiene) or COT (1,3,5-cyclooctatriene) may prevent the formation of such particles [41,42]. Thus, we assume that COD allows ruthenium to better penetrate the pores of the aromatic polymer due to the formation of a Ru–COD complex, which allows us to obtain small nanoparticles by the acid sites after the reduction.

**Figure 4:**
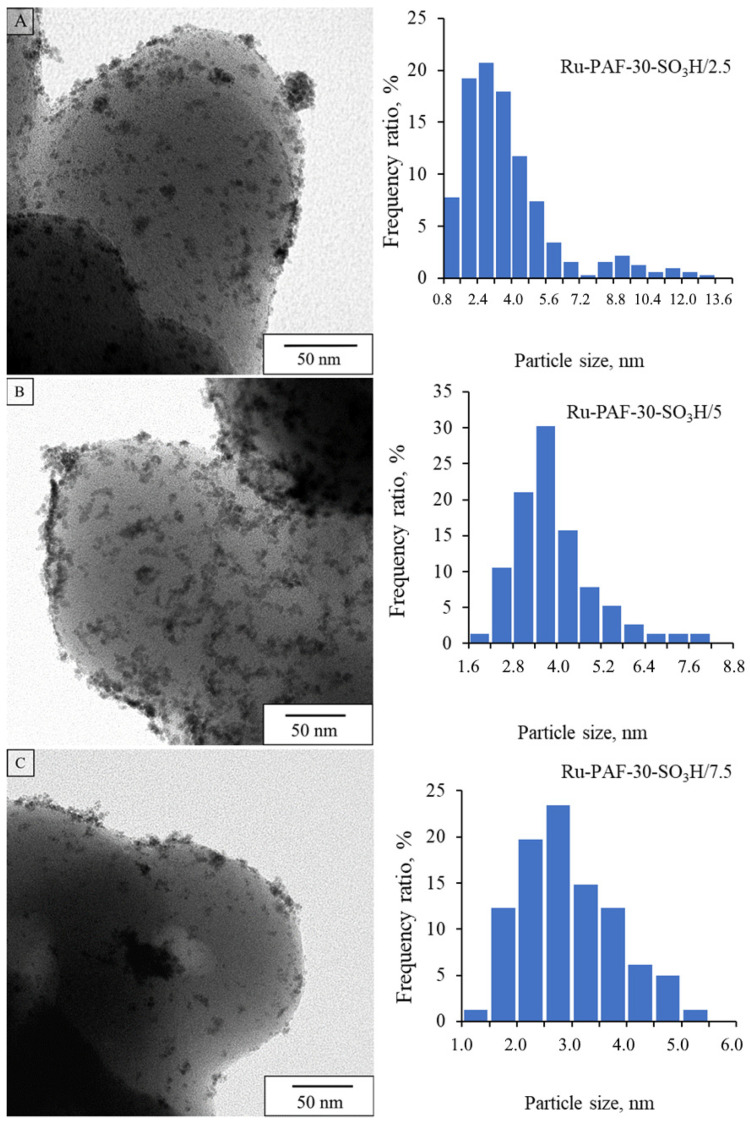
Transmission electron microscopy (TEM) microphotographs and particle size distribution for Ru-PAF-30-SO_3_H/2.5 (**A**), Ru-PAF-30-SO_3_H/5 (**B**), and Ru-PAF-30-SO_3_H/7.5 (**C**).

TEM data for all three catalysts of Series A demonstrate ruthenium nanoparticles with average sizes of 2.6 nm, 3.7 nm, and 2.8 nm for Ru-PAF-30-SO_3_H/2.5, Ru-PAF-30-SO_3_H/5, and Ru-PAF-30-SO_3_H/7.5, respectively, unevenly distributed over the surface of PAFs (Figure 4). A large number of agglomerates of these nanoparticles was also detected for all three samples. The content of ruthenium in the catalysts was 1.47, 4.67, and 0.5 wt.% for Ru-PAF-30-SO_3_H/2.5, Ru-PAF-30-SO_3_H/5, and Ru-PAF-30-SO_3_H/7.5, respectively. Apparently, this method does not allow metal to be deposited into pores effectively, as most of the ruthenium was located on the polymer surface.

**Figure 5:**
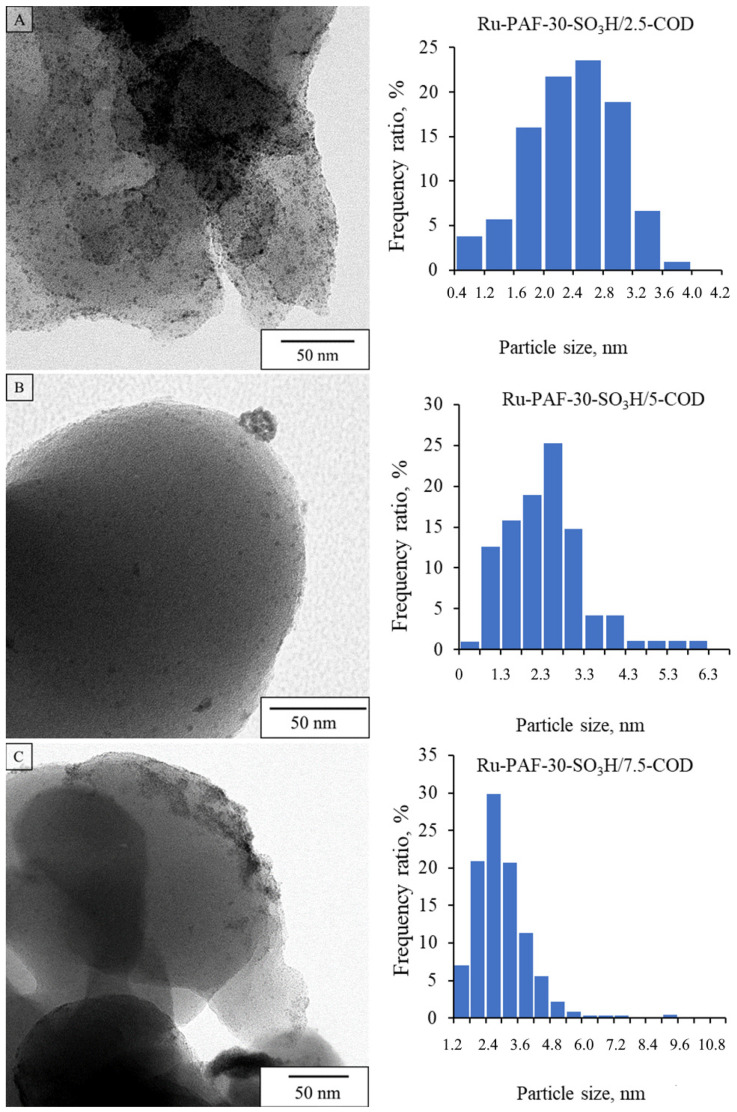
TEM microphotographs and particle size distribution for Ru-PAF-30-SO_3_H/2.5-COD (**A**), Ru-PAF-30-SO_3_H/5-COD (**B**), and Ru-PAF-30-SO_3_H/7.5-COD (**C**).

According to TEM data, for catalysts of Series B synthesized in the presence of 1,5-cyclooctadiene, particle size distribution depended heavily on a content of sulfo groups in the support (Figure 5). For Ru-PAF-30-SO_3_H/2.5-COD, small nanoparticles with an average size of 2.2 nm were mainly localized in the pore structure; agglomerates of nanoparticles were not detected. TEM microphotographs for Ru-PAF-30-SO_3_H/5-COD, with a higher concentration of -SO_3_H groups, demonstrated fewer nanoparticles with a slightly larger average size of 2.5 nm, also mainly localized in the pore structure. However, the formation of agglomerates on the surface of the support was also noted. For Ru-PAF-30-SO_3_H/7.5-COD catalyst with an even greater amount of sulfo groups, a further decrease in the number of nanoparticles as well as an increase in the proportion of agglomerates was observed due to the hindrance of metal diffusion into pores. The surface area of all catalysts declined after metal deposition, but in the case of catalysts synthesized using of 1,5-cyclooctadiene, the area decreased more significantly, even despite the lower metal content in the catalysts (Figure S3, Table 2). In addition, a significant decrease in the surface area of the Ru-PAF-30-SO_3_H/7.5 catalyst may indicate immobilization of ruthenium in the pores of the support or blocking of the pores by metal agglomerates.

The catalyst’s synthesis using 1,5-cyclooctadiene generally results in the formation of smaller particles. The average size of nanoparticles for samples synthesized without cyclooctadiene (series A) was 2.7–3.8 nm as opposed to 2.2–2.8 nm for samples synthesized in the presence of cyclooctadiene (series B). It should be noted, however, that metal content in samples of series B did not exceed 0.8 wt.%.

**Figure 6:**
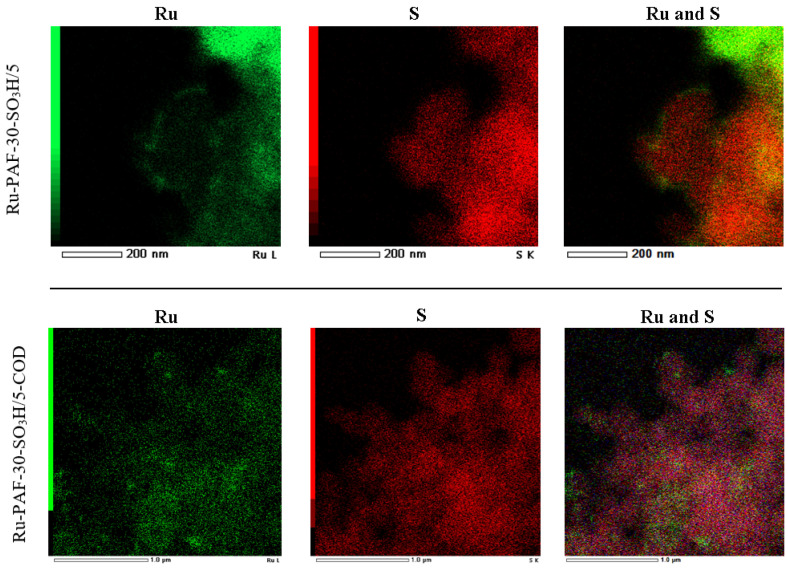
EDX mapping results for Ru-PAF-30-SO_3_H/5 and Ru-PAF-30-SO_3_H/5-COD.

Thus, COD makes it possible to immobilize ruthenium closer to sulfo groups, whereas, in its absence, ruthenium is predominantly concentrated on the surface of PAF particles and further from acid sites. This can be clearly observed in the EDX microphotographs (Figure 6): in the case of the Ru-PAF-30-SO_3_H/5 catalyst, ruthenium and sulfur are spatially separated. At the same time, in the case of the Ru-PAF-30-SO_3_H/5-COD catalyst, ruthenium and sulfur are located closer to each other and their distribution in the material is more uniform.

Survey XPS spectra (Figure S4) confirm the presence of C, O, S, and Ru and the absence of Na in analyzed catalysts (Table S1). The analysis of the S2p spectra of catalysts shows no significant changes compared to the spectra of initial PAFs (Figure S5). At present, it is not possible to make a conclusion about the interaction of a -SO_3_H groups with supported ruthenium. However, there are some interesting differences between the two types of catalysts (synthesized with and without COD).

**Figure 7:**
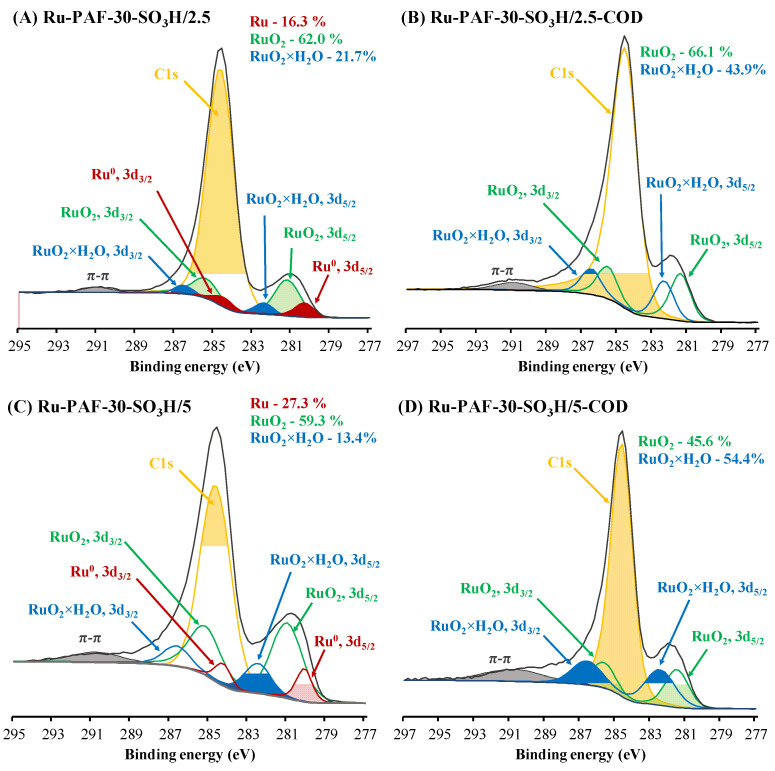

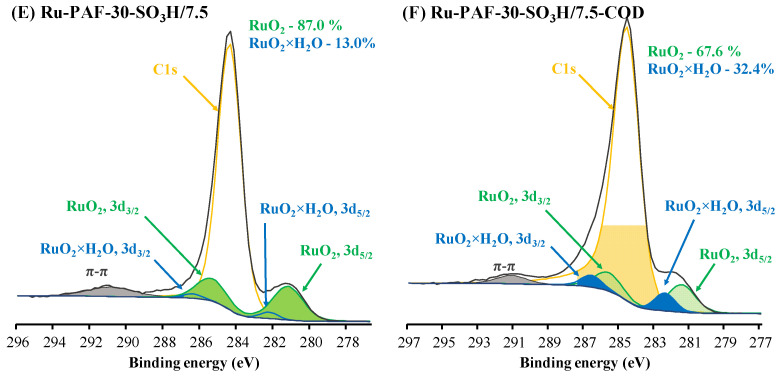
High-resolution XPS spectrum of C1s and Ru3d region.

The resolved spectra of ruthenium (Figure 7) demonstrate three Ru-3d doublets (3d_5/2_, 3d_3/2_, Δ = 4.17 eV) with the binding energies of Ru 3d_5/2_ of ~280.1–281.2, ~281.2–281.4, and ~282.3–282.4 eV assigned to the metallic Ru^0^, RuO_2_, and RuO_2_ × H_2_O states, respectively (Table S2) [43]. Ru-PAF-30-SO_3_H/2.5 and Ru-PAF-30-SO_3_H/5 catalysts contain ruthenium in all of the chemical states listed above, and Ru-PAF-30-SO_3_H/7.5 contains approximately 87% of RuO_2_ and 13% of RuO_2_×H_2_O. The presence of the Ru^0^ phase in the Ru-PAF-30-SO_3_H/2.5 and Ru-PAF-30-SO_3_H/5 catalysts can be explained by the higher metal content and larger nanoparticle size in them, while in the Ru-PAF-30-SO_3_H/7.5 catalyst, all ruthenium is oxide state due to its lower content, the smaller size of nanoparticles, and their location predominantly on the surface of the support. At the same time, the catalysts synthesized using COD do not contain Ru^0^ at all, and the ratio RuO_2_ × H_2_O/RuO_2_ is higher. We believe that, in fact, the fraction of RuO_2_ × H_2_O state is lower and the binding energies for both RuO_2_ × H_2_O and RuO_2_ states are higher due to the proximity of ruthenium nanoparticles to -SO_3_H groups and interaction with them. However, due to the lack of exact electron binding energies, it is difficult to accurately deconvolve the spectra. Nevertheless, the difference between the XPS spectra of the two types of catalysts is clearly visible.

### 3.2. Catalytic Tests

Transformations of three model lignin-based bio-oil compounds—guaiacol, catechol, and veratrole—were studied using the obtained catalysts. J. Shangguan et al. [44] suppose that hydrodeoxygenation of guaiacol involves the formation of quasi-equilibrium intermediates as a result of the addition of several H-atoms, which then may undergo various transformations. These routes may occur either concomitantly or in sequence via multiple pathways. Hydrogen addition to the benzene ring results in the formation of 2-methoxycyclohexanol, hydrogenolysis of C_ar_–OH bond produces anisole, while hydrogenolysis of C_ar_–OC_alk_ bond—phenol, and the one of C_ar_O–C_alk_ bond—catechol. Moreover, the primary products may also undergo further hydrodeoxygenation, producing cycloalkanes and cycloalkanols. This reaction scheme is very complex and poses significant challenges in studying the reaction pathways, so substrate transformations have been considered using simplified schemes to assess the impact of quantity and location of acid groups.

#### 3.2.1. Investigation of the Influence of the Number of Acid Sites

**Figure 8:**
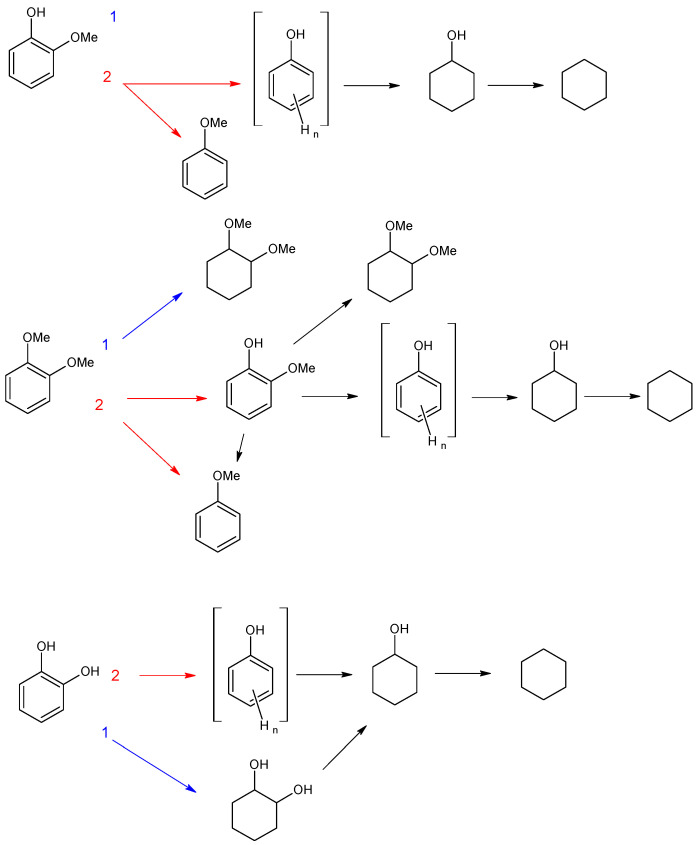
Suggested schemes of HDO of guaiacol, veratrole and catechol on the Ru-PAF-30-SO_3_H/2.5, Ru-PAF-30-SO_3_H/5 and Ru-PAF-30-SO_3_H/7.5 catalysts.

For catalytic systems synthesized in this work, two main HDO pathways can be observed for guaiacol (Figure 8): (1) hydrogenation of the aromatic ring and (2) C_ar_–OC_alk_ cleavage followed by hydrogenation of resulting phenolic adduct [45]. For Ru-PAF-30-SO3H/2.5, the reaction proceeds predominantly along path (1) with the formation of 2-methoxycyclohexanol (56%), and the deoxygenation process (2) proceeds only until the formation of cyclohexanol (26%). The product of complete HDO, cyclohexane, is observed only in trace amounts (<2%). Most likely, the amount of acid sites in the catalyst is not enough to provide C_alk_–O cleavage from the saturated ring, so path (1) turns out to be preferable. Since, after saturation of the aromatic ring, further transformations become unlikely, the formation of the hydrodeoxygenation product, cyclohexanol, apparently follows path (2), and 2-methoxycyclohexanol does not undergo further transformations. Also, according to the literature data, the breaking of C_ar_–O bond on Ru metal centers is easier than the C_alk_–O one [46,47].

Since Ru-PAF-30-SO_3_H/5 catalyst contains more sulfo groups that promote the cleavage of the C_ar_–O bond, the amount of phenolic adduct in the reaction products increases [48], and due to the ease of its hydrogenation, the yield of cyclohexanol also increases [47]. In addition, the amount of sulfo groups in the catalyst becomes sufficient to carry out deoxygenation reactions, so the resulting cyclohexanol is further converted to cyclohexane via deoxygenation and hydrogenation [49]. With a further increase in the concentration of acid sites in the catalysts, cyclohexane becomes the main reaction product, but the conversion decreases. Thus, guaiacol HDO over Ru-PAF-30-SO_3_H/7.5 gives cyclohexane with 63% selectivity, but overall conversion was only 30% (Table 3). Apparently, due to the low metal loading and high -SO_3_H/Ru ratio in Ru-PAF-30-SO_3_H/7.5 catalyst, the rate of the hydrogenation reaction decreases, while rates of acid-promoted reactions become high, all hydrogenated products undergo further deoxygenation.

The conversion pathways for veratrol were similar to those observed for guaiacol: (1) hydrogenation of substrate to dimethoxycyclohexane, and (2) conversion to guaiacol followed by hydrogenation as described above, or demethoxylation to anisole. For catalyst Ru-PAF-30-SO_3_H/2.5, hydrogenation route (1) also turns out to be the most preferable, as in the case of guaiacol, and the main products were dimethoxycyclohexane (66%) and 2-methoxycyclohexanol (22%). As the number of acid sites in the catalyst increased, acid-promoted reactions (deoxygenation, isomerization) become preferable. Thus, the yields of dimethoxycyclohexane and 2-methoxycyclohexanol in veratrole HDO over Ru-PAF-30-SO_3_H/5 were 46% and 7%, respectively, while the yields of cyclohexanol and cyclohexane increased to 18%. With a further increase in the number of acid groups in the catalyst Ru-PAF-30-SO_3_H/7.5, the elimination of methoxy group from the saturated ring became even more prevalent, which resulted in the significant growth of the cyclohexane yield (57%).

In the case of catechol, either (1) hydrogenation of the substrate to 1,2-dihydroxycyclohexane occurs or (2) its deoxygenation to phenolic adduct, followed by further hydrogenation to cyclohexanol (Figure 8). For the catalyst Ru-PAF-30-SO_3_H/2.5, as in the case of two previous substrates, route (1) is preferred, giving 1,2-dihydroxycyclohexane with 47% yield. However, the yield of the deoxygenation product (cyclohexanol) is significantly higher (24%) compared to the other substrates. The reason of this phenomenon is most likely the strong donor effect of the -OH group, since it provides a much lower barrier for C–O hydrogenolysis [50]. HDO of catechol over Ru-PAF-30-SO_3_H/5 gives cyclohexanol as a main product (73%), while the yield of dihydroxycyclohexanol decreases to 13% Also, an increase in the number of acid sites in the catalyst contributes the cleavage of C_alk_-O bond with the formation of cyclohexane (12%). However, conversion of catechol drastically decreases when Ru-PAF-30-SO_3_H/7.5 was used as the catalyst. Unlike veratrole, the catechol molecule contains two -OH groups, which participate in the adsorption of the molecule on the surface of nanoparticles. Probably, due to this feature, adsorption of the catechol molecule requires more space on the surface of nanoparticles, and there may not be centers with the suitable size and geometry [44].

#### 3.2.2. Investigation of the Influence of the Mutual Arrangement of Metal and Acid Centers

**Figure 9:**
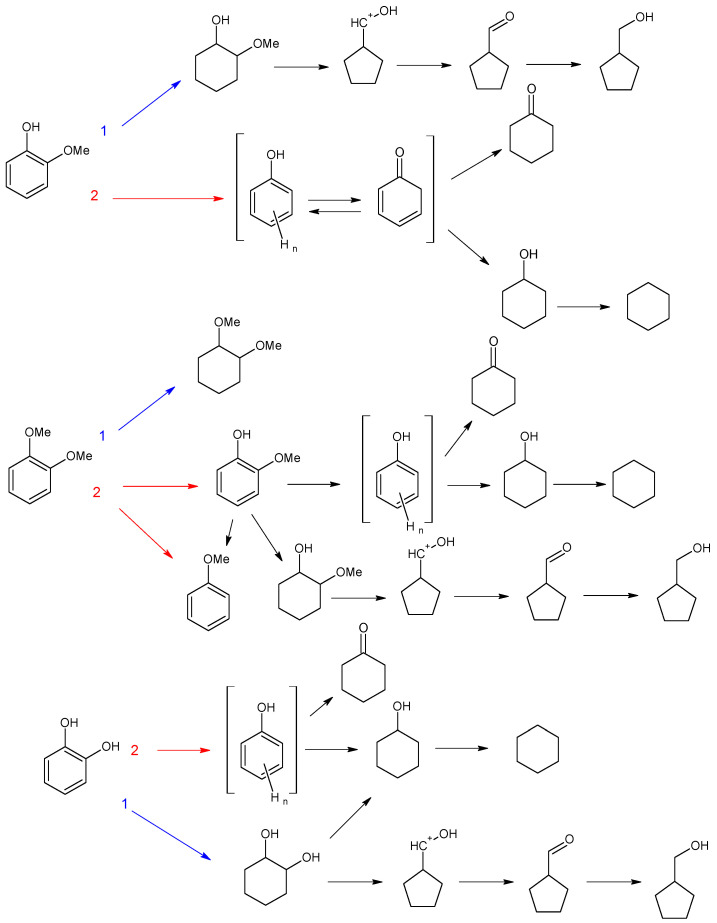
Suggested schemes of HDO of guaiacol, veratrole and catechol on the Ru-PAF-30-SO_3_H/2.5-COD, Ru-PAF-30-SO_3_H/5-COD and Ru-PAF-30-SO_3_H/7.5-COD catalysts.

Catalysts synthesized using 1,5-COD have also been studied in the HDO of guaiacol, catechol and veratrole (Table 4, Figure 9). There are no significant differences in the composition of guaiacol HDO products with Ru-PAF-30-SO_3_H/2.5 and Ru-PAF-30-SO_3_H/2.5-COD catalysts, synthesized without and with 1,5-COD, respectively. The structure of the products remained the same (2-methoxycyclohexanol, cyclohexanol, cyclohexane), however, their ratio differed. Thus, the yield of 2-methoxycyclohexanol decreased (33% vs. 56% for the Ru-PAF-30-SO_3_H/2.5 catalyst), while the yield of cyclohexanol was higher (40% vs. 26% for the Ru-PAF-30-SO_3_H/2.5 catalyst). Differences in the product distribution may be due to the smaller average size of metal nanoparticles in the Ru-PAF-30-SO_3_H/2.5-COD catalyst (2.2 nm vs. 2.9 nm for the Ru-PAF-30-SO_3_H/2.5 catalyst), which leads to higher activity of the catalyst in HDO [51]. In the case of veratrole, the structure of the reaction products also remained the same, and their concentrations also differed. Thus, the yield of cyclohexane increased to 13% (vs. 3% for the Ru-PAF-30-SO_3_H/2.5 catalyst), while the yields of 1,2-dimethoxycyclohexane and 2-methoxycyclohexanol were lower (26% and 10% respectively). However, in the case of catechol, significant changes in the activity of the Ru-PAF-30-SO_3_H/2.5-COD catalyst and in the composition of the reaction products were observed. While the conversion of catechol on Ru-PAF-30-SO_3_H/2.5 was 99%, and the main products were 1,2-dihydroxycyclohexane (47%) and cyclohexanol (24%), reaction on Ru-PAF-30-SO_3_H/2.5-COD gives cyclohexanol with only 20% yield and 2-isopropoxyphenol (7%) as a side reaction product.

The Ru-PAF-30-SO_3_H/7.5-COD catalyst did not show activity in the hydrogenation of substrates, despite approximately the same ruthenium content as the Ru-PAF-30-SO_3_H/7.5 catalyst. Considering the significantly smaller surface area of this catalyst compared to both the original support and the Ru-PAF-30-SO_3_H/7.5 catalyst (Table 2), it is possible that the obtained results can be associated with difficulties in the diffusion of substrate molecules to ruthenium inside the catalyst pores due to the high concentration of sulfo groups.

Quite interesting results were obtained for the Ru-PAF-30-SO_3_H/5-COD catalyst. Despite the low metal content, the conversion of all three substrates on this catalyst was high (more than 70%). However, for all the substrates studied, the formation of carbonyl compounds (cyclohexanone and cyclopentanecarbaldehyde) in significant quantities was observed. We assume that this phenomenon may be associated with a fairly close arrangement of metal nanoparticles and Brønsted acid sites: sulfo groups interact with carbonyl compounds resulting from partial hydrogenation of substrates, deactivating the carbonyl group and preventing its further hydrogenation [52]. Most likely, under such conditions, desorption of the ketone becomes more preferable than its further hydrogenation. Selectivity to the carbonyl compounds, as well as conversion of substrates, decreases in the series catechol-guaiacol-veratrole.

#### 3.2.3. Catalysts Recycling

The recyclability of the catalysts was also tested using Ru-PAF-30-SO_3_H/5 and Ru-PAF-30-SO_3_H/5-COD catalysts as an example (Table 5). In both cases, a decrease in the activity of the catalysts is observed. Possible reasons for the decrease in catalyst activity include leaching of ruthenium, the blockage of catalyst pores of with heavy molecules, and hydrolysis of sulfo-groups [53]. In case of the Ru-PAF-30-SO_3_H/5 catalyst, the yield of cyclohexane significantly decreases from 55 to 11%, whereas the yield of cyclohexanol increases slightly. Also, the presence of a small amount of heavy products was noticed after the third run. The Ru-PAF-30-SO_3_H/5-COD also loses its activity—however, the yield of all products, including alkylation products, decreases. To establish the reasons for the decrease in activity, the catalysts were studied after the 3^rd^ cycle of tests using XPS, TEM and FTIR.

**Figure 10:**
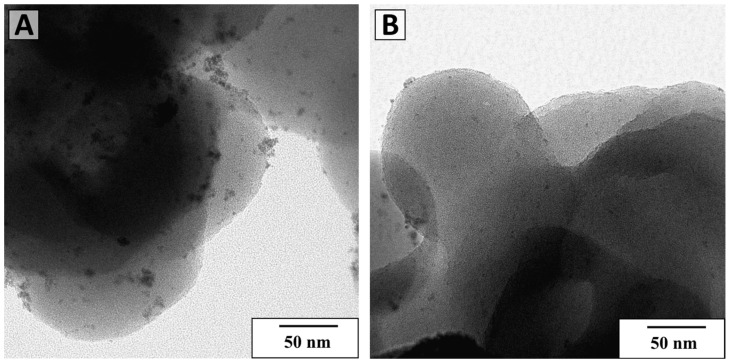
TEM microphotographs for Ru-PAF-30-SO_3_H/5 (**A**) and Ru-PAF-30-SO_3_H/5-COD (**B**) after 3rd catalytic cycle.

**Figure 11:**
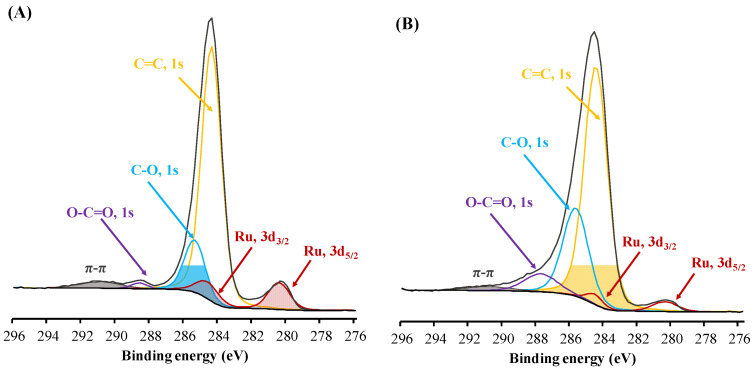
High-resolution XPS spectrum of C1s and Ru3d region for Ru-PAF-30-SO_3_H/5 (**A**) and Ru-PAF-30-SO_3_H/5-COD (**B**) after 3rd catalytic cycle.

According to the TEM (Figure 10), both catalysts contain Ru nanoparticles after 3^rd^ catalytic cycle. However, their number, especially in the case of Ru PAF 30-SO_3_H/5 catalyst, has become significantly smaller. According to ICP-AES, the metal content in the catalysts decreased and became 1.31 and 0.41 wt. % for Ru PAF 30-SO_3_H/5 and Ru-PAF-30-SO_3_H/5-COD, respectively. XPS spectra of C1s and Ru3d lines (Figure 11) show that ruthenium in both catalysts is completely reduced and is present in the Ru(0) phase. In addition, the presence of new components, presumably C-O and O-C=O, is observed in the spectra of both catalysts. Also, the intensity of these components is higher in the case of the Ru-PAF-30-SO_3_H/5-COD catalyst. The position of the S2p lines remains the same (Figure S6), and new signals do not appear.

**Figure 12:**
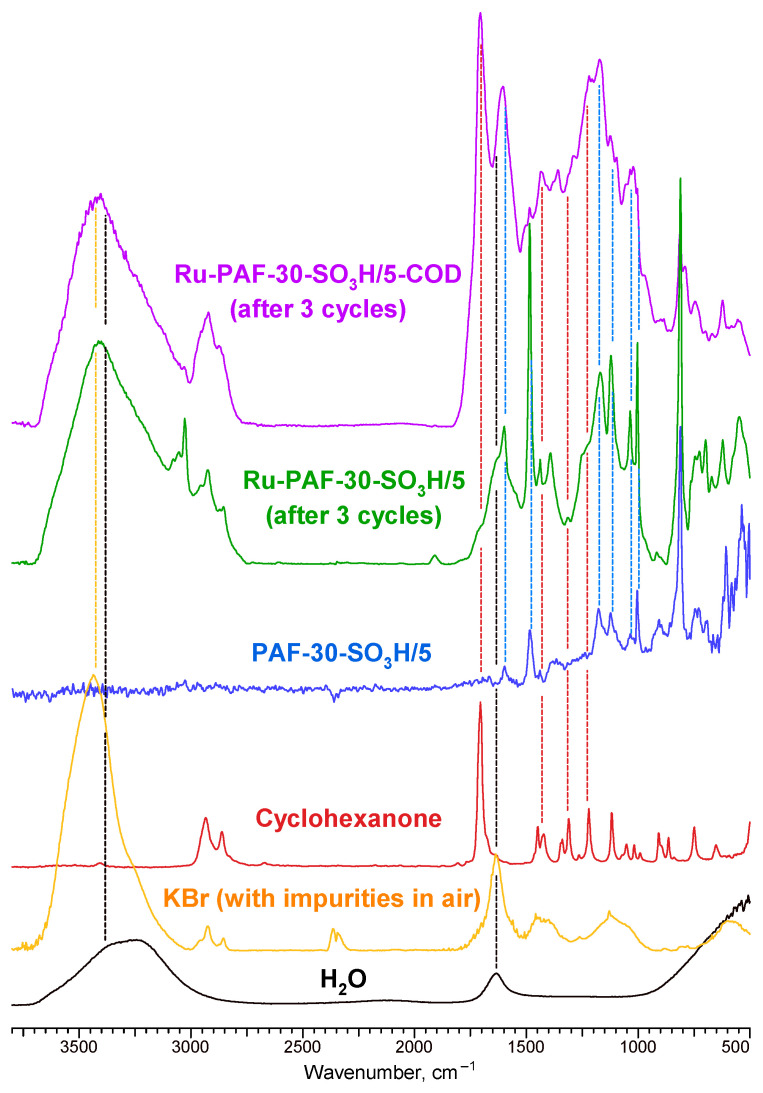
FTIR spectra of catalysts after 3rd catalytic cycle, PAF-30-SO_3_H/5, cyclohexanone, H_2_O and KBr (with impurities in air).

Finally, the recycled catalysts were investigated using FTIR (Figure 12). In the case of both catalysts, the absorption bands characteristic of the original support was preserved. However, a significant change in the appearance of the FTIR spectra can also be observed. Most likely, this is due to the formation of condensation products of guaiacol and its hydrogenation products inside the pores of the catalyst, which also correlates with the results of XPS spectroscopy. However, it is interesting to evaluate the difference in the spectra of the catalysts. In the case of the Ru-PAF-30-SO_3_H/5-COD catalyst, the pores apparently contain products of cyclohexanone condensation, as indicated by the presence of characteristic absorption bands (the most indicative—1704 cm^−1^, 2800–2990 cm^−1^). In the case of Ru-PAF-30-SO_3_H/5 catalyst, these absorption bands are also present, but their intensity is much lower. At the same time, the FTIR spectrum contains new adsorption bands with maxima at 1393, 3028–3080, 1250, and 1903 cm^−1^, which may relate to derivatives of aromatic compounds. Unfortunately, even roughly establishing their composition seems quite problematic.

The catalytic performance of the synthesized catalysts was compared with other ruthenium catalysts based on both inorganic and carbon-based supports (Table S3). The synthesized catalysts exhibit in some cases similar activity, and in some cases, they are superior to the catalysts described in the literature. For instance, Ru-PAF-30-SO_3_H-5/COD was more effective and selective in cyclohexanone formation than Ru/HY catalyst, and Ru-PAF-30-SO_3_H/2.5-COD catalyst possessed the comparable activity with Ru-CARF catalyst even in more mild conditions.

## 4. Conclusions

In summary, porous aromatic frameworks modified with different concentration of sulfo groups were used to synthesize two series of Ru catalysts: series A—without the addition of 1,5-cyclooctadiene (COD), and series B—with the addition of 1,5-cyclooctadiene. The materials were characterized by FTIR, TEM, XPS, low temperature N_2_ adsorption and elemental analysis. It has been shown that the use of COD in the synthesis of catalysts (series B) makes it possible to obtain smaller metal nanoparticles located closer to acid sites, whereas series A catalysts contain Ru nanoparticles on the surface of the support. The catalysts of were active in HDO of guaiacol, veratrole and catechol, and the composition of reaction products depended on the method of synthesis of the catalyst and the selected support. Thus, increase in the number of acid groups in the catalysts enhances their activity in deoxygenation processes, but can be dramatically decrease their hydrogenation activity due to the blocking of metal sites by -SO_3_H groups. Also, in the case of the Ru-PAF-30-SO_3_H/5-COD catalyst, synthesized using COD, the main reaction products for all substrates were carbonyl compounds, which can be explained by the close location of metal nanoparticles and Brønsted acid sites in the catalyst. The catalysts can be used for at least three cycles, but they gradually lose activity as a result of leaching of ruthenium and the formation of high molecular weight products in the pores.

## Figures and Tables

**Table 1:** Textural properties, sulfur content, and acidity of the materials.

Material	S_BET_, m^2^/g	Total Pore Volume, cm^3^/g	Sulfur Content, wt. %	Acidity, mmol/g
PAF-30	484	0.28	-	-
PAF-30-SO_3_H/2.5	427	0.19	2.5	0.82
PAF-30-SO_3_H/5	369	0.09	5	1.64
PAF-30-SO_3_H/7.5	197	0.08	7.5	2.34

**Table 2:** Ruthenium content, average sizes of Ru nanoparticles in synthesized catalysts and their surface area.

Catalyst	d_av_, nm	Ru Content, wt.%	S/Ru, mol:mol	S_BET_, m^2^/g
Ru-PAF-30-SO_3_H/2.5	2.8	1.47	5.36	410
Ru-PAF-30-SO_3_H/5	3.8	4.67	3.47	359
Ru-PAF-30-SO_3_H/7.5	2.7	0.50	47.28	153
Ru-PAF-30-SO_3_H/2.5-COD	2.2	0.79	9.98	388
Ru-PAF-30-SO_3_H/5-COD	2.5	0.76	20.73	354
Ru-PAF-30-SO_3_H/7.5-COD	2.8	0.47	50.29	94

**Table 3:** Conversion and product yields of HDO of guaiacol, catechol and veratrole over Ru catalysts synthesized without 1,5-COD (Series A).

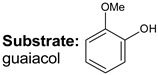	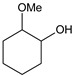		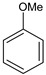		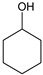		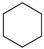		Conversion, %
* Ru-PAF-30	63		—		34		—		97
Ru-PAF-30-SO_3_H/2.5	56		—		26		2		84
Ru-PAF-30-SO_3_H/5	35		2		8		55		100
Ru-PAF-30-SO_3_H/7.5	—		4		7		19		30
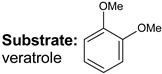	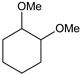	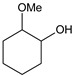		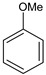	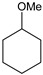	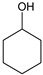	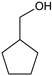	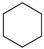	Conversion, %
* Ru-PAF-30	95	1		—	—	3	—	—	99
Ru-PAF-30-SO_3_H/2.5	66	22		—	7	2	—	3	100
Ru-PAF-30-SO_3_H/5	46	7		—	6	18	2	18	97
Ru-PAF-30-SO_3_H/7.5	2	2		2	—	—	11	57	78
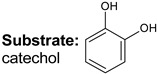	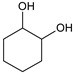	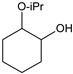		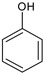	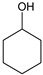	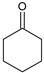	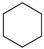	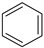	Conversion, %
* Ru-PAF-30	58	—		1	41	—	—	0.2	100
Ru-PAF-30-SO_3_H/2.5	47	13		4	24	1	8	2	99
Ru-PAF-30-SO_3_H/5	13	—		—	73	1	12	—	99
Ru-PAF-30-SO_3_H/7.5	—	—		—	—	5	—	—	5

Reaction conditions: 250 °C; 2 h; 3 MPa H_2_; 500 μL water; 5 mg catalyst; and 0.38 mmol substrate; * to assess the effect of sulfo groups, the catalysts were additionally compared with the catalyst synthesized before [47].

**Table 4:** Conversion and product yields of HDO of guaiacol, catechol, and veratrole over Ru catalysts synthesized with 1,5-COD (Series B).

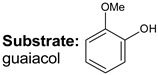	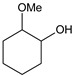	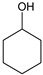	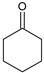		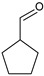	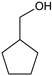	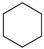	Alkylation products		Conversion, %
Ru-PAF-30-SO_3_H/2.5-COD	33	40	—		—	—	16	—		89
Ru-PAF-30-SO_3_H/5-COD	—	—	44		8	11	1	19		83
Ru-PAF-30-SO_3_H/7.5-COD	—	—	—		—	—	—	—		0
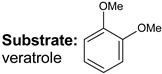	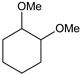	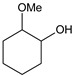	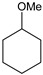	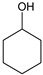	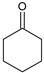	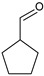	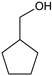	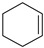	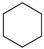	Conversion, %
Ru-PAF-30-SO_3_H/2.5-COD	26	10	7	6	—	—	—	—	13	62
Ru-PAF-30-SO_3_H/5 -COD	3	—	—	—	18	2	7	11	38	79
Ru-PAF-30-SO_3_H/7.5-COD	—	—	—	—	—	—	—	—	—	0
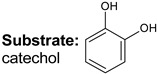	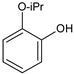	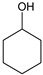	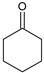		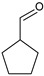		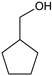	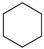		Conversion, %
Ru-PAF-30-SO_3_H/2.5-COD	7	20	—		—		1	1		29
Ru-PAF-30-SO_3_H/5 -COD	10	—	70		15		5	—		100
Ru-PAF-30-SO_3_H/7.5-COD	—	—	—		—		—	—		0

Reaction conditions: 250 °C; 2 h; 3 MPa H_2_; 500 μL water; 5 mg catalyst; and 0.38 mmol substrate.

**Table 5:** Conversion and product yields of guaiacol hydrogenation in recycling experiments with Ru-PAF-30-SO_3_H/5 and Ru-PAF-30-SO_3_H/5-COD catalysts.

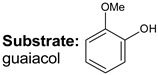		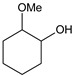	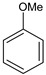		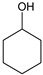	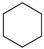		Alkylation products	Conversion, %
Ru-PAF-30-SO_3_H/5	Run 1	35	2		8	55		—	100
	Run 2	39	—		12	35		—	86
	Run 3	27	—		14	11		6	58
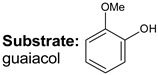		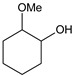	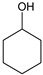	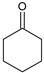	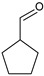	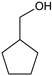	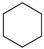	Alkylation products	Conversion, %
Ru-PAF-30-SO_3_H/5 -COD	Run 1	—	—	44	8	11	1	19	83
	Run 2	4	2	31	2	—	1	11	52
	Run 3	2	2	12	—	—	2	8	28

Reaction conditions: 250 °C; 2 h; 3 MPa H_2_; 500 μL water; 5 mg catalyst; and 0.38 mmol guaiacol.

## Data Availability

Data are contained within the article and Supplementary Materials.

## Supplementary Materials

The following supporting information can be downloaded at: https://www.mdpi.com/article/10.3390/polym15234618/s1, Figure S1: The XPS survey spectra for PAF-30-SO_3_H/X (X = 2.5, 5, 7.5); Figure S2: High-resolution XPS spectra of S2p region for PAF-30-SO_3_H/X (X = 2.5, 5, 7.5); Figure S3: N_2_ adsorption isotherms for synthesized catalysts; Figure S4: The XPS survey spectra for ruthenium catalysts; Figure S5: High-resolution XPS spectra of S2p region; Figure S6: High-resolution XPS spectra of S2p region for Ru-PAF-30-SO_3_H/5 (A) and Ru-PAF-30-SO_3_H/5 (B) catalysts after the 3^rd^ catalytic run; Table S1: Components of the XPS spectra; Table S2: Peak parameters for XPS spectra of obtained ruthenium catalysts; Table S3: Guaiacol HDO over different catalysts. Refs. [54,55,56,57,58,59,60] are cited in the Supplementary Material.
