# Supplementary material for: Hydrodeoxygenation of Lignin-Based Compounds over Ruthenium Catalysts Based on Sulfonated Porous Aromatic Frameworks
Source: Polymers (Basel). 2023 Dec 4;15(23):4618. doi: 10.3390/polym15234618 (PMC10708665; doi:10.3390/polym15234618)
Supplement: Supplementary file 1 [file polymers-15-04618-s001.zip › polymers-2665824-supplementary.pdf]

Supplementary Material

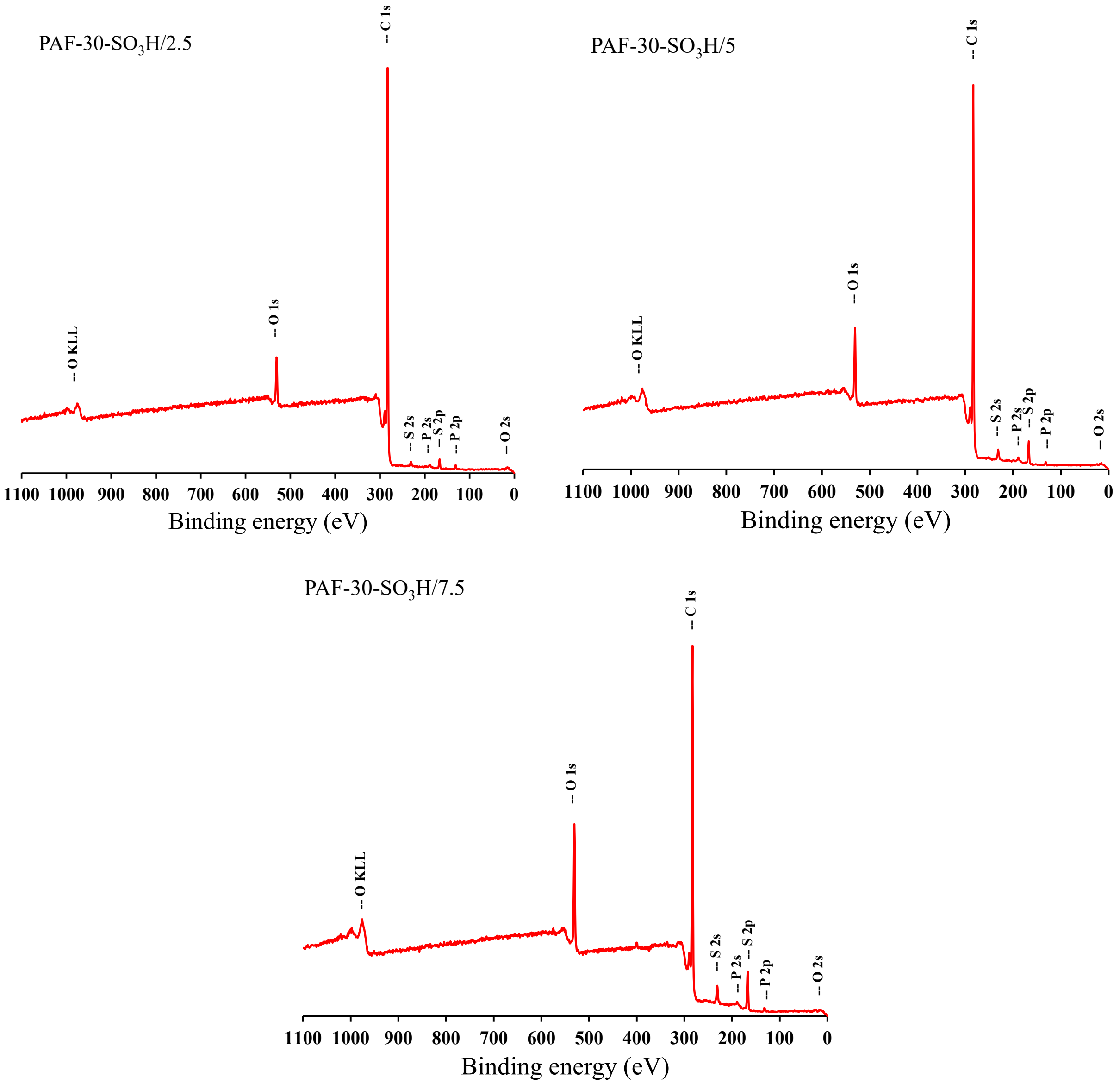

Figure S1. The XPS survey spectra for PAF-30-SO_3H/X (X = 2.5, 5, 7.5).

Table S1. Components of the XPS spectra.

Materials	Element content, at. %				
	C	*O*	*P*	*S*	*Ru*
PAF-30-SO_3H/2.5	92.0	6.4	0.6	1.0	-
PAF-30-SO_3H/5	87.1	10.1	0.5	2.3	-
PAF-30-SO_3H/7.5	80.9	14.6	0.6	3.9	-
Ru-PAF-30-SO_3H/2.5-COD	79.8	15.3	0.2	0.6	4.1
Ru-PAF-30-SO_3H/5-COD	76.2	17.2	0.3	3.3	3.0
Ru-PAF-30-SO_3H/7.5-COD	77.0	17.1	0.2	3.4	2.3
Ru-PAF-30-SO_3H/2.5	79.7	15.1	0.3	0.5	4.4
Ru-PAF-30-SO_3H/5	71.5	19.2	0.3	2.7	6.3
Ru-PAF-30-SO_3H/7.5	76.5	16.9	0.1	3.5	3.0

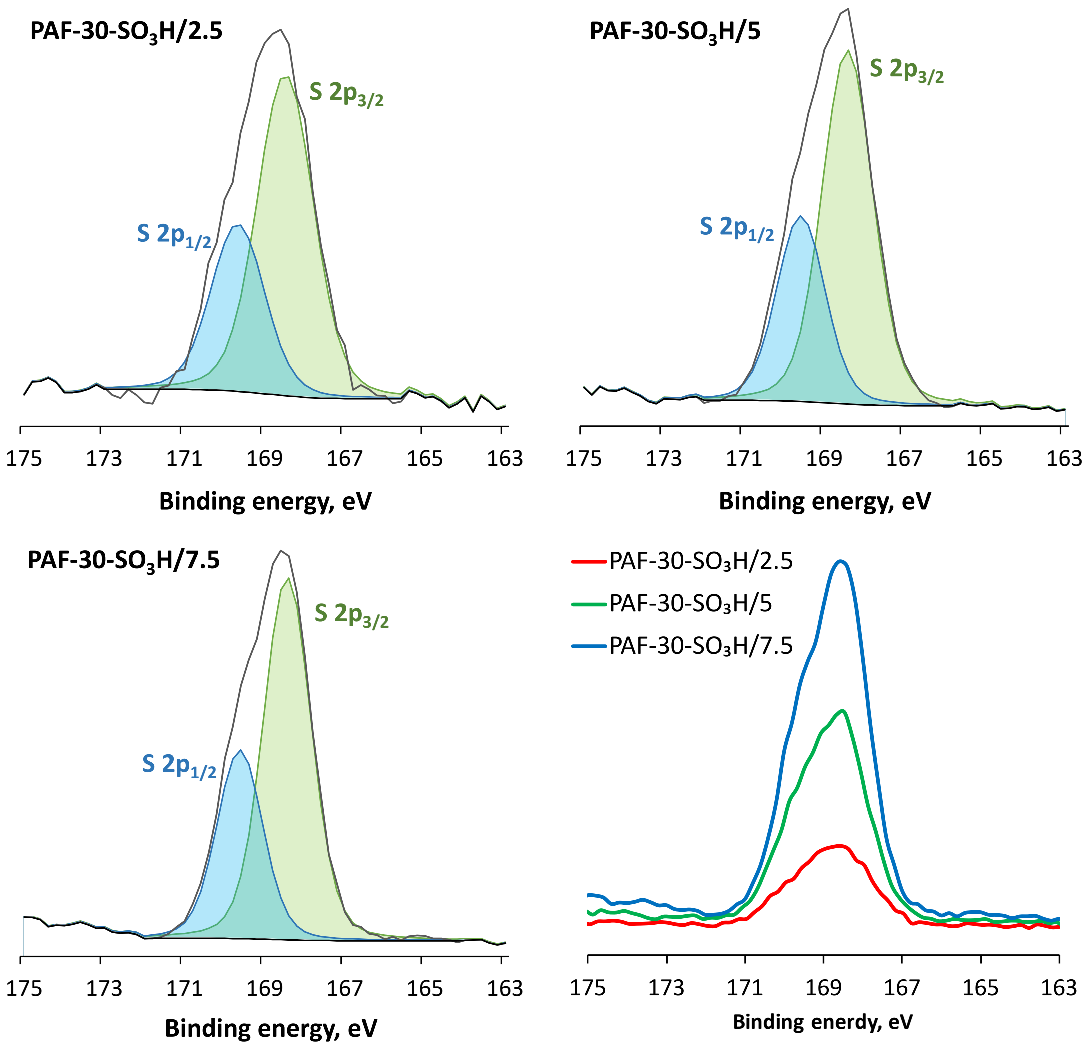

Figure S2. High-resolution XPS spectra of S2p region for PAF-30-SO_3H/X (X = 2.5, 5, 7.5).

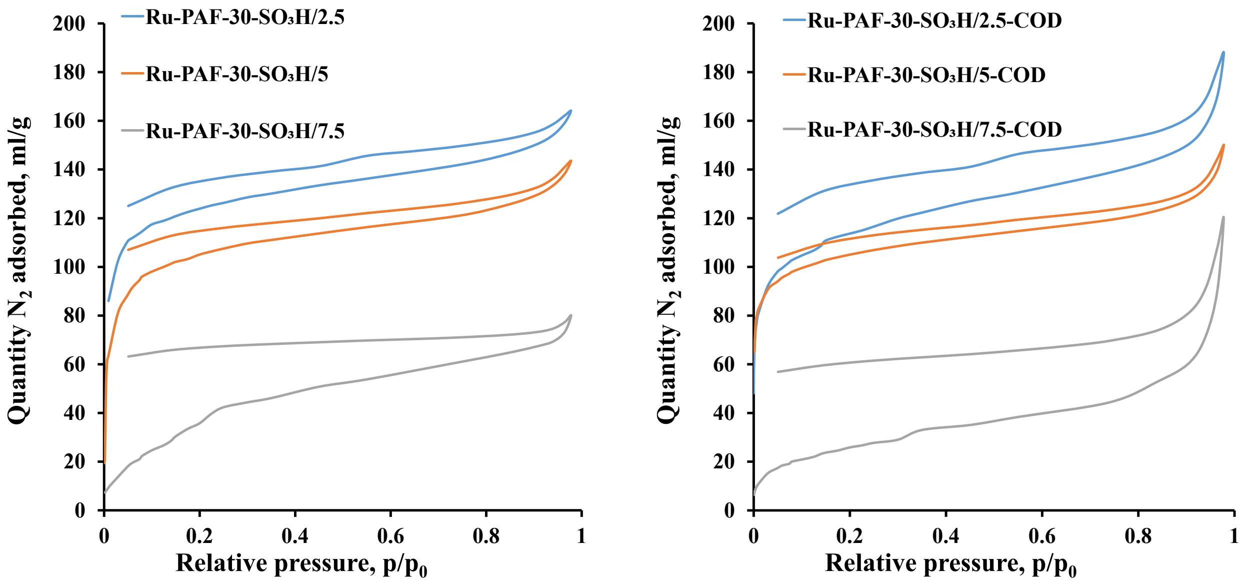

Figure S3. N_2 adsorption isotherms for synthesized catalysts.

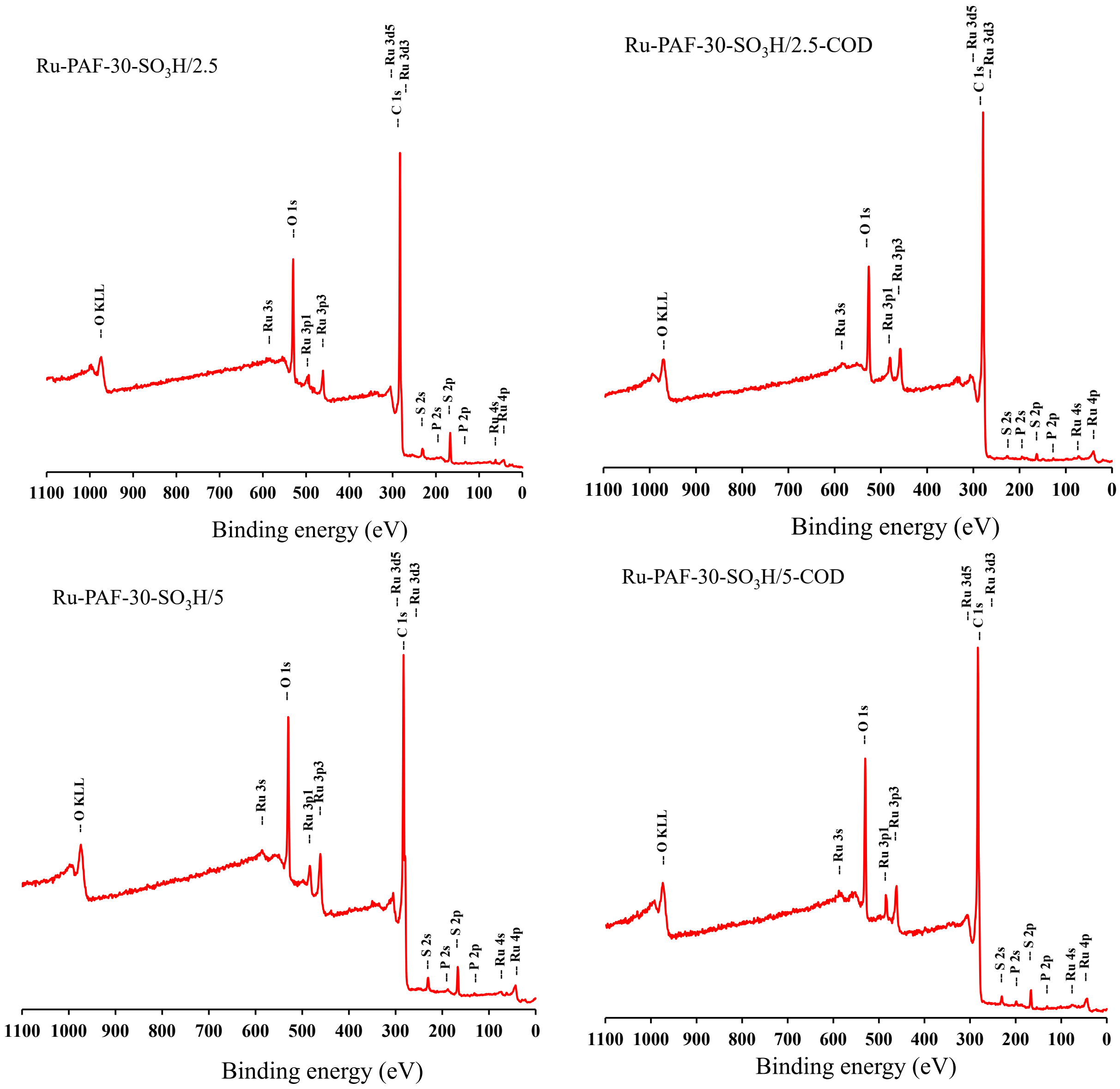

Figure S4. The XPS survey spectra for ruthenium catalysts.

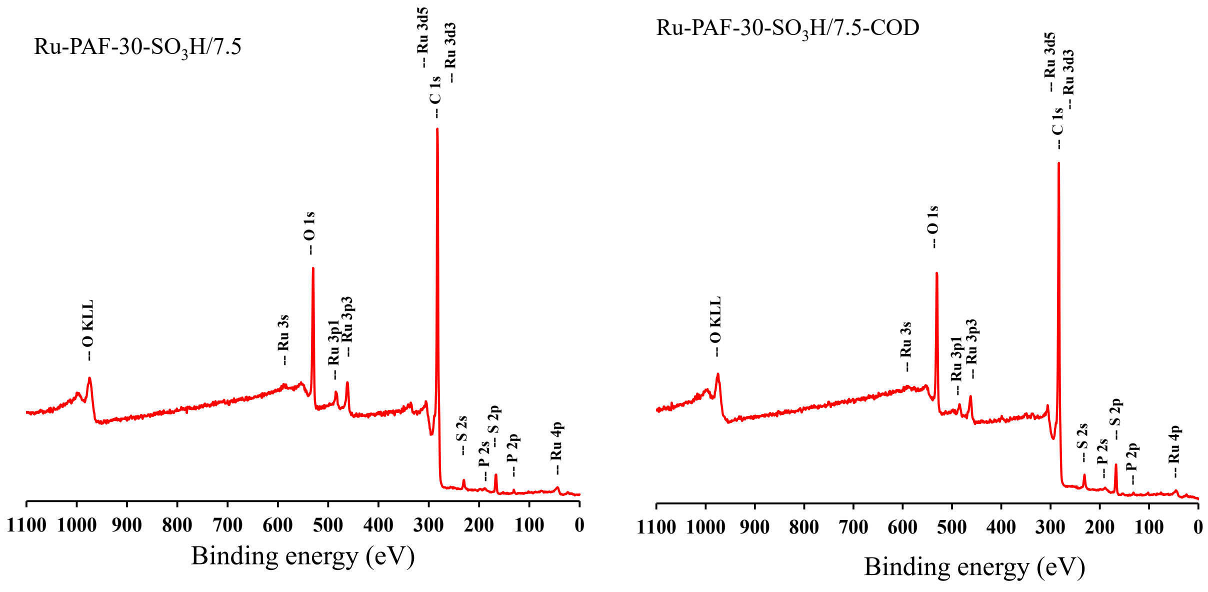

Figure S4. (end) The XPS survey spectra for ruthenium catalysts

Table S2. Peak parameters for XPS spectra of obtained ruthenium catalysts.

Catalyst	Ru^0		RuO_2		$RuO_2 \times xH_2O$		C	π-π
	3d 5/2	3d 3/2	3d 5/2	3d 3/2	3d 5/2	3d 3/2	1s	1s
Ru-PAF-30-SO_3H / 2.5	**16.3%**		**62.0%**		**21.7%**		284.58	290.86
	280.19	284.36	281.10	285.27	282.30	286.47		
Ru-PAF-30-SO_3H / 5	**27.3%**		**59.3%**		**13.4%**		284.60	290.80
	280.10	284.27	281.17	285.34	282.46	286.63		
Ru-PAF-30-SO_3H / 7.5	–		**87.0%**		**13.0%**		284.54	290.98
			281.36	285.53	282.40	286.57		
Ru-PAF-30-SO_3H / 2.5-COD	–		**66.1%**		**43.9%**		284.52	290.95
			281.37	285.54	282.31	286.48		
Ru-PAF-30-SO_3H / 5-COD	–		**45.6%**		**54.4%**		284.50	290.80
			281.40	285.57	282.36	286.53		
Ru-PAF-30-SO_3H / 7.5-COD	–		**67.6%**		**32.4%**		284.54	291.00
			281.39	285.56	282.34	286.51		

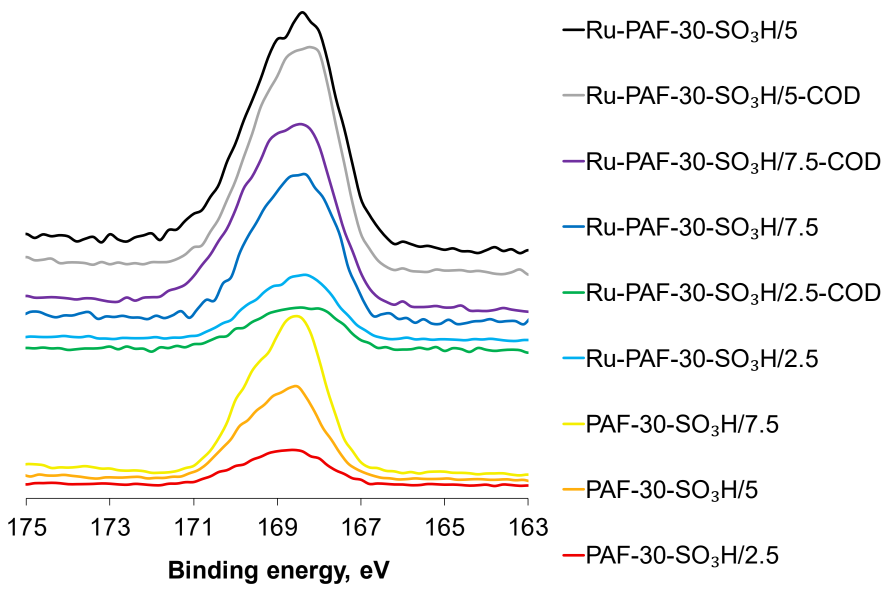

Figure S5. High-resolution XPS spectra of S2p region.

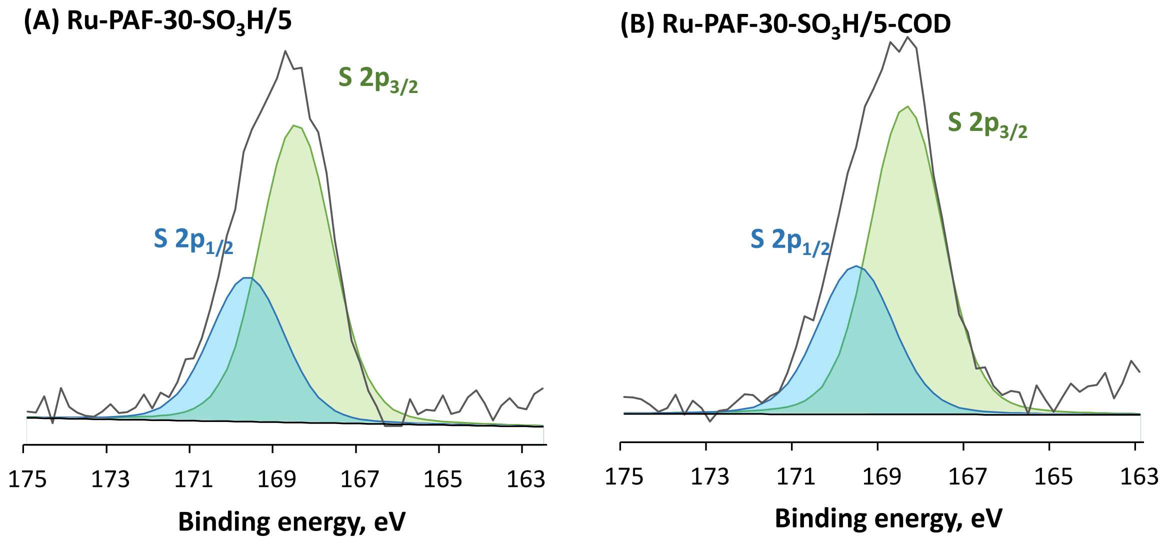

Figure S6. High-resolution XPS spectra of S2p region for Ru-PAF-30-SO_3H/5 (A) and Ru-PAF-30-SO_3H/5 (B) catalysts after the 3rd catalytic run.

Table S3. Guaiacol HDO over different catalysts.

Catalyst	Sub/Me	Conv., %	Products	Yield, %	Ref.
Ru-PAF-30-SO_3H/2.5-COD<br>(Ru = 0.79 wt.%)<br>3 MPa H_2; 250 °C;<br>500 μL H_2O; 0.38 mmol guaiacol;<br>5 mg of catalyst; 2 h;	972	89	2-methoxy-cyclohexanol<br>Cyclohexanol<br>Cyclohexane	33<br>40<br>16	This work
Ru NPs@SILP-1.00<br>(Ru = 0.32 wt.%)<br>12 MPa H_2; 175 °C;<br>1 mL decalin; 2.4 mmol guaiacol;<br>75 mg of catalyst; 16 h	1000	>99%	2-methoxy-cyclohexanol<br>Methoxycyclohexane<br>Cyclohexanol<br>Cyclohexane	14<br>4<br>1<br>81	[1]
Ru-MWCNT<br>(Ru = 5 wt.%)<br>4 MPa H_2, 270 °C;<br>29 mL H_2O, 8,96 mmol guaiacol;<br>20 mg of catalyst; 1h	906	98.1 %	Cyclohexane<br>Methoxycyclohexane<br>Cyclohexanol<br>Cyclohexanone<br>Cyclopentanemethanol<br>2-methoxy-cyclohexanol<br>1,2-Cyclohexanediol<br>Catechol	34.9<br>0.2<br>2.2<br>0.1<br>2.6<br>13.6<br>0.3<br>0.1	[2]
Ru-AC<br>(Ru = 5 wt.%)<br>4 MPa H_2, 270 °C;<br>29 mL H_2O, 8,96 mmol guaiacol;<br>20 mg of catalyst; 1h	906	96.1 %	Cyclopentane<br>Cyclohexane<br>Methylcyclohexane<br>Methoxycyclohexane<br>Cyclohexanol<br>Cyclohexanone<br>Cyclopentanemethanol<br>Anisole<br>Phenol<br>2-methoxy-cyclohexanol<br>1,2-Cyclohexanediol<br>Catechol	0.2<br>24.7<br>0.3<br>0.4<br>10.2<br>0.8<br>1.6<br>0.2<br>0.3<br>22.7<br>1.0<br>0.1	[2]
Ru-CARF<br>(Ru = 5 wt.%)<br>4 MPa H_2, 270 °C;<br>29 mL H_2O, 8,96 mmol guaiacol;<br>20 mg of catalyst; 1h	906	81.6 %	Cyclohexane<br>Methylcyclohexane<br>Methoxycyclohexane<br>Cyclohexanol<br>Cyclohexanone<br>Cyclopentanemethanol<br>Anisole<br>Phenol<br>2-methoxy-cyclohexanol<br>1,2-Cyclohexanediol<br>Catechol	5.7<br>1.1<br>0.1<br>24.5<br>1.9<br>0.7<br>0.3<br>4.2<br>17.3<br>0.5<br>0.1	[2]

Ru-Vulcan<br>(Ru = 5 wt.%)<br>4 MPa H_2, 270 °C;<br>29 mL H_2O, 8,96 mmol guaiacol;<br>20 mg of catalyst; 1h	906	77.0 %	Cyclohexane<br>Methylcyclohexane<br>Methoxycyclohexane<br>Cyclohexanol<br>Cyclohexanone<br>Cyclopentanemethanol<br>Anisole<br>Phenol<br>2-methoxy-cyclohexanol<br>1,2-Cyclohexanediol<br>Catechol	11.9<br>1.2<br>0.1<br>12.7<br>1.1<br>0.6<br>2.2<br>12.3<br>8.8<br>0.7<br>0.1	[2]
Ru-PAF-30-SO_3H/5-COD<br>(Ru = 0.76 wt.%)<br>3 MPa H_2; 250 °C;<br>500 μL H_2O; 0.38 mmol guaiacol;<br>5 mg of catalyst; 2 h;	1010	83%	Cyclohexanone<br>Cyclopentanemethanol<br>Cyclopentanecarbaldehyde<br>Cyclohexane<br>Alkylation products	44<br>11<br>8<br>1<br>19	This work
Ru/HY<br>(Ru = 5 wt.%)<br>4 MPa H_2; 250 °C;<br>30 mL H_2O; 0.81 mmol guaiacol;<br>100 mg of catalyst; 2 h;	16	91	Cyclohexanone<br>Cyclohexane<br>Cyclohexanol<br>Gases<br>Ring-open products<br>Cyclopentylmethanol<br>Dimers<br>Others	27.5<br>18.5<br>18.1<br>10.0<br>8.2<br>7.9<br>6.3<br>3.5	[3]
Ru-PAF-30-SO_3H/5<br>(Ru = 4.68 wt.%)<br>3 MPa H_2; 250 °C;<br>500 μL H_2O; 0.38 mmol guaiacol;<br>5 mg of catalyst; 2 h;	164	100	2-methoxy-cyclohexanol<br>Anisole<br>Cyclohexanol<br>Cyclohexane	35<br>2<br>8<br>55	This work
NSMP-Ru<br>(Ru = 3.6 wt.%)<br>5 MPa H_2, 200 °C;<br>1 mL H_2O, 0.4 mmol guaiacol;<br>5 mg of catalyst, 2h	225	>99 %	Methoxycyclohexanol<br>Cyclohexanol<br>Methylcyclohexanol	86<br>13<br><1	[4]
5%Ru/AMWCNTs<br>(Ru = 5 wt.%)<br>2MPa H_2, 200 °C;<br>5.4 mL decalin, 10 mmol guaiacol;<br>100 mg of catalyst, 200 min	202	49.32 %	Cyclohexanol<br>1,2-Cyclohexanediol<br>Cyclohexane<br>Cyclohexanone<br>Phenol<br>Benzene<br>Others	33.2<br>5.2<br>1.4<br>1.7<br>1.2<br>1.8<br>4.9	[5]
Ru/HNT-t (3)<br>(Ru = 2 wt.%)<br>3 MPa H_2; 180 °C;<br>2.7 mL H_2O; 2.42 mmol guaiacol;<br>61 mg of catalyst; 3 h;	200	100%	Cyclohexanol<br>2-methoxycyclohexanol<br>4-methylcyclohexanol<br>Cyclohexane<br>Phenol<br>p-cresol<br>Cyclohexanone	35.4<br>25.4<br>24.1<br>12.6<br>2.0<br>0.4<br>0.1	[6]

$Ru/TiO_2–Al_2O_3$ (Ru = 1.04 wt.%) 1 MPa H_2; 240 °C; 20 mL octane; 0.81 mmol guaiacol; 50 mg of catalyst; 4 h;	157	91.4 %	Cyclohexane<br>Cyclohexanol<br>2-methoxycyclohexanol	89.4<br>9.0<br>1.6	[7]

References

1. Luska, K. L.; Migowski, P.; El Sayed, S.; Leitner, W. Synergistic Interaction within Bifunctional Ruthenium Nanoparticle/SILP Catalysts for the Selective Hydrodeoxygenation of Phenols. *Angewandte Chemie International Edition* **2015**, *54* (52), 15750–15755. https://doi.org/10.1002/ANIE.201508513.
2. Dwiatmoko, A. A.; Zhou, L.; Kim, I.; Choi, J. W.; Suh, D. J.; Ha, J. M. Hydrodeoxygenation of Lignin-Derived Monomers and Lignocellulose Pyrolysis Oil on the Carbon-Supported Ru Catalysts. *Catal Today* **2016**, *265*, 192–198. https://doi.org/10.1016/J.CATTOD.2015.08.027.
3. Wang, H.; Ruan, H.; Feng, M.; Qin, Y.; Job, H.; Luo, L.; Wang, C.; Engelhard, M. H.; Kuhn, E.; Chen, X.; Tucker, M. P.; Yang, B. One-Pot Process for Hydrodeoxygenation of Lignin to Alkanes Using Ru-Based Bimetallic and Bifunctional Catalysts Supported on Zeolite Y. *ChemSusChem* **2017**, *10* (8), 1846–1856. https://doi.org/10.1002/CSSC.201700160.
4. Boronoev, M. P.; Shakirov, I. I.; Ignat'eva, V. I.; Maximov, A. L.; Karakhanov, E. A. A Nanospherical Mesoporous Ruthenium-Containing Polymer as a Guaiacol Hydrogenation Catalyst. *Petroleum Chemistry* **2019**, *59* (12), 1300–1306. https://doi.org/10.1134/S096554411912003X.
5. Long, W.; Liu, P.; Xiong, W.; Hao, F.; Luo, H. Conversion of Guaiacol as Lignin Model Component Using Acid-Treated, Multi-Walled Carbon Nanotubes Supported Ru–MnO Bimetallic Catalysts. *Can J. Chem.* **2020**, *98* (2), 57–65. https://doi.org/10.1139/CJC-2019-0261.
6. Zasypalov, G.; Vutolkina, A.; Klimovsky, V.; Abramov, E.; Vinokurov, V.; Glotov, A. Hydrodeoxygenation of Guaiacol over Halloysite Nanotubes Decorated with Ru Nanoparticles: Effect of Alumina Acid Etching on Catalytic Behavior and Reaction Pathways. *Appl Catal B* **2024**, *342*, 123425. https://doi.org/10.1016/J.APCATB.2023.123425.
7. Lin, B.; Li, R.; Shu, R.; Wang, C.; Yuan, Z.; Liu, Y.; Chen, Y. Synergistic Effect of Highly Dispersed Ru and Moderate Acid Site on the Hydrodeoxygenation of Phenolic Compounds and Raw Bio-Oil. *Journal of the Energy Institute* **2020**, *93* (3), 847–856. https://doi.org/10.1016/j.joei.2019.07.009.

Disclaimer/Publisher's Note: The statements, opinions and data contained in all publications are solely those of the individual author(s) and contributor(s) and not of MDPI and/or the editor(s). MDPI and/or the editor(s) disclaim responsibility for any injury to people or property resulting from any ideas, methods, instructions or products referred to in the content.
